# A framework of biomarkers for vascular aging: a consensus statement by the Aging Biomarker Consortium

**DOI:** 10.1093/lifemedi/lnad033

**Published:** 2023-08-30

**Authors:** Aging Biomarker Consortium, Le Zhang, Jun Guo, Yuehong Liu, Shimin Sun, Baohua Liu, Qi Yang, Jun Tao, Xiao-Li Tian, Jun Pu, Huashan Hong, Miao Wang, Hou-Zao Chen, Jie Ren, Xiaoming Wang, Zhen Liang, Yuan Wang, Kai Huang, Weiqi Zhang, Jing Qu, Zhenyu Ju, Guang-Hui Liu, Gang Pei, Jian Li, Cuntai Zhang

**Affiliations:** Department of Geriatrics, Tongji Hospital, Tongji Medical College, Huazhong University of Science and Technology, Wuhan 430030, China; Key Laboratory of Vascular Aging, Ministry of Education, Tongji Hospital, Tongji Medical College, Huazhong University of Science and Technology, Wuhan 430030, China; The Key Laboratory of Geriatrics, Beijing Institute of Geriatrics, Institute of Geriatric Medicine, Chinese Academy of Medical Sciences, Beijing Hospital/National Center of Gerontology of National Health Commission, Beijing 100730, China; Department of Radiology, Beijing Chaoyang Hospital, Capital Medical University, Beijing 100020, China; Institute of Molecular Cell Biology, Center for Molecular Biomedicine, Jena University Hospital, Jena 07743, Germany; School of Basic Medical Sciences, Shenzhen University Medical School, Shenzhen 518055, China; Department of Radiology, Beijing Chaoyang Hospital, Capital Medical University, Beijing 100020, China; Department of Hypertension and Vascular Disease, The First Affiliated Hospital, Sun-Yat-sen University, Guangzhou 510080, China; Aging and Vascular Diseases, Human Aging Research Institute (HARI) and School of Life Science, Nanchang University, and Jiangxi Key Laboratory of Human Aging, Nanchang 330031, China; Division of Cardiology, State Key Laboratory of Systems Medicine for Cancer, Renji Hospital, School of Medicine, Shanghai Jiao Tong University, Shanghai Cancer Institute, Shanghai 200127, China; Department of Geriatrics, Fujian Key Laboratory of Vascular Aging, Fujian Medical University Union Hospital, Fuzhou 350001, China; State Key Laboratory of Cardiovascular Disease, Fuwai Hospital, National Center for Cardiovascular Diseases, Chinese Academy of Medical Sciences and Peking Union Medical College, Beijing 100037, China; Clinical Pharmacology Center, Fuwai Hospital, National Center for Cardiovascular Diseases, Chinese Academy of Medical Sciences and Peking Union Medical College, Beijing 100037, China; Department of Biochemistry & Molecular Biology, State Key Laboratory of Common Mechanism Research for Major Diseases, Institute of Basic Medical Sciences, Chinese Academy of Medical Sciences & Peking Union Medical College, Beijing 100005, China; Medical Epigenetics Research Center, Chinese Academy of Medical Sciences, Beijing 100005, China; CAS Key Laboratory of Genomic and Precision Medicine, Beijing Institute of Genomics, Chinese Academy of Sciences and China National Center for Bioinformation, Beijing 100101, China; Department of Geriatrics, Xijing Hospital, Air Force Medical University, Xi’an 710032, China; Shenzhen People’s Hospital, Shenzhen 518020, China; Beijing Anzhen Hospital, Capital Medical University, Beijing 100029, China; Clinic Center of Human Gene Research, Union Hospital, Tongji Medical College, Huazhong University of Science and Technology, Wuhan 430022, China; Hubei Key Laboratory of Metabolic Abnormalities and Vascular Aging, Huazhong University of Science and Technology, Wuhan 430022, China; Hubei Clinical Research Center of Metabolic and Cardiovascular Disease, Huazhong University of Science and Technology, Wuhan 430022, China; Department of Cardiology, Union Hospital, Tongji Medical College, Huazhong University of Science and Technology, Wuhan 430022, China; CAS Key Laboratory of Genomic and Precision Medicine, Beijing Institute of Genomics, Chinese Academy of Sciences and China National Center for Bioinformation, Beijing 100101, China; State Key Laboratory of Stem Cell and Reproductive Biology, Institute of Zoology, Chinese Academy of Sciences, Beijing 100101, China; Key Laboratory of Regenerative Medicine of Ministry of Education, Institute of Aging and Regenerative Medicine, Jinan University, Guangzhou 510632, China; University of Chinese Academy of Sciences, Beijing 100049, China; State Key Laboratory of Membrane Biology, Institute of Zoology, Chinese Academy of Sciences, Beijing 100101, China; Institute for Stem Cell and Regeneration, Chinese Academy of Sciences, Beijing 100101, China; Collaborative Innovation Center for Brain Science, School of Life Science and Technology, Tongji University, Shanghai 200092, China; The Key Laboratory of Geriatrics, Beijing Institute of Geriatrics, Institute of Geriatric Medicine, Chinese Academy of Medical Sciences, Beijing Hospital/National Center of Gerontology of National Health Commission, Beijing 100730, China; Department of Geriatrics, Tongji Hospital, Tongji Medical College, Huazhong University of Science and Technology, Wuhan 430030, China; Key Laboratory of Vascular Aging, Ministry of Education, Tongji Hospital, Tongji Medical College, Huazhong University of Science and Technology, Wuhan 430030, China

## Abstract

Aging of the vasculature, which is integral to the functioning of literally all human organs, serves as a fundamental physiological basis for age-related alterations as well as a shared etiological mechanism for various chronic diseases prevalent in the elderly population. China, home to the world’s largest aging population, faces an escalating challenge in addressing the prevention and management of these age-related conditions. To meet this challenge, the Aging Biomarker Consortium of China has developed an expert consensus on biomarkers of vascular aging (VA) by synthesizing literature and insights from scientists and clinicians. This consensus provides a comprehensive assessment of biomarkers associated with VA and presents a systemic framework to classify them into three dimensions: functional, structural, and humoral. Within each dimension, the expert panel recommends the most clinically relevant VA biomarkers. For the functional domain, biomarkers reflecting vascular stiffness and endothelial function are highlighted. The structural dimension encompasses metrics for vascular structure, microvascular structure, and distribution. Additionally, proinflammatory factors are emphasized as biomarkers with the humoral dimension. The aim of this expert consensus is to establish a foundation for assessing the extent of VA and conducting research related to VA, with the ultimate goal of improving the vascular health of the elderly in China and globally.

## Introduction

Dr Thomas Sydenham (1624–89), known as “The English Hippocrates”, once remarked, “A man is as old as his arteries.” This profound statement from the renowned physician and author of the celebrated medical textbook “Observationes Medicae” continues to resonate even after four centuries. This prompts us to question the true age of our arteries and the vasculature as a whole. Despite the accumulation of a vast spectrum of biological measurements, we still lack a comprehensive and systematic assessment of biomarkers for vascular aging (VA) (Table 1). Therefore, we find ourselves in an ongoing pursuit to discover the most effective metric for this purpose.

**Table 1.** Abbreviations in this consensus

COR	Class of recommendation
LOE	Level of evidence
VA	Vascular aging
ABC	Aging Biomarker Consortium
4D-FLOW MRI	Four-dimensional flow magnetic resonance imaging
8-Oxo-dGsn	8-Oxo-7,8-dihydrodeoxyguanosine
8-OxoGsn	8-Oxo-7,8-dihydroguanosin
ABI	Ankle brachial index
ASL	Arterial spin labelling
baPWV	Brachial-ankle pulse wave velocity
BOLD-fMRI	Blood oxygenation level dependent functional magnetic resonance imaging
CACS	Coronary artery calcium score
CAVI	Cardio-ankle vascular index
CBF	Cerebral blood flow
CBV	Cerebral blood volume
CCVD	Cerebro-cardiovascular disease
cfPWV	Carotid-femoral pulse wave velocity
CFR	Coronary flow reserve
cIMT	Carotid intima-media thickness
CMVD	Coronary microvascular disease
CTA	Computed tomography angiography
CVD	Cardiovascular disease
CVR	Cerebrovascular reserve
DNAmAge	DNA methylation age
DNMT3α	DNA methyltransferase 3 alpha
DP-pCASL	Diffusion-prepared pseudo-continuous arterial spin labelling
DSA	Digital substruction angiography
ECM	Extracellular matrix
EMPs	Endothelial microparticles
EPCs	Endothelial progenitor cells
ePWV	Estimated pulse wave velocity
FE	Fundoscopic examination
FGF21	Fibroblast growth factor 21
FHS	The Framingham Heart Study
FMD	Flow-mediated dilation
GP	Glycan peak
GWAS	The Genome-Wide Association Studies
HERVK	Human endogenous retroviruse K
HMDB	Human metabolome database
hs-CRP	High-sensitivity C-reactive protein
IFNγ	Interferon γ
IGF-1	Insulin-like growth factor 1
IgG	Immunoglobulin G
IL-1	Interleukin-1
IL-1Ra	Interleukin-1 receptor antagonist
IL-6	Interleukin-6
IL-8	Interleukin-8
IMR	Index of microcirculatory resistance
KDM	The Klemera and Doubal method
LC-MS/MS	High-performance liquid chromatography-triple quadrupole mass spectrometry
LINE-1	Long interspersed element-1
lncRNA	Long noncoding RNA
LSCI	Laser speckle contrast imaging
MBP	Mean blood pressure
MCI	Mild cognitive impairment
MESA	Multi-Ethnic Study of Atherosclerosis
miRNA	microRNA
MMP-2	Matrix metallopeptidase 2
MPI	Myocardial perfusion imaging
MRA	Magnetic resonance angiography
MRI	Magnetic resonance imaging
ncRNA	Noncoding RNA
NHANES	National Health and Nutrition Examination Survey
NMR	Nuclear magnetic resonance
OCT	Optical coherence tomography
OEF	Oxygen extraction fraction
ox-LDL	Oxidized low-density lipoprotein
PAD	Peripheral arterial disease
PC	Phase-contrast
PP	Pulse pressure
PUFAs	Polyunsaturated fatty acids
PWV	Pulse wave velocity
QSM	Quantitative susceptibility mapping
RDW	Red blood cell distribution width
RHI	Reactive hyperemia index
ROS	Reactive oxygen species
SASP	Senescence-associated secretory phenotype
SCORE	Systematic Coronary Risk Evaluation
SNAP	Simultaneous noncontrast angiography and intraplaque hemorrhage
SPRINT	Systolic Blood Pressure Intervention Trial
SWI	Susceptibility weighted imaging
TMAO	Trimethylamine N-oxide
TNFα	Tumor necrosis factor alpha
TOF-MRA	Time-of-flight magnetic resonance angiography
ufPWV	Ultrafast ultrasound imaging pulse wave velocity
VCAM-1	Vascular cell adhesion molecule-1
VECs	Vascular endothelial cells
VEGF	Vascular endothelial growth factor
VSMCs	Vascular smooth muscle cells
VWMRI	Vessel wall magnetic resonance imaging

The vasculature is a complex and diverse organ comprising a wide array of components, including arteries, microvessels, veins, and specialized vessels such as hepatic sinusoids, the blood–brain barrier, and alveolar blood–gas barriers. Arteries play a crucial role in transporting oxygen and nutrient-rich blood from the heart, while microvessels facilitate the distribution of oxygen and nutrients to all tissues and remove waste products, such as carbon dioxide and metabolic byproducts, from the organs. Veins, on the other hand, are responsible for returning oxygen-depleted blood back to the heart following the elimination of waste products through organs such as the lungs and kidneys [1]. Structurally, large arteries and veins consistently exhibit a trilaminar configuration that includes the *adventitia*, *media*, and *intima*. In contrast, microvessels typically consist of a uniform monolayer of endothelial cells adhering to the basement membrane, facilitating efficient tissue oxygenation and the exchange of substances between the blood and tissues [1].

VA encompasses a range of morphological and functional changes that occur in blood vessels as a natural consequence of the aging process. Functionally, VA is characterized by increased vascular stiffness, altered responsiveness to vasoactive factors, decreased capacity for vascular neovascularization, and increased secretion of inflammatory factors, among other changes [2–4]. Morphologically, arterial aging is characterized by thickening of the *intima*-*media*, disruption, and disarray of elastic fibers, increased deposition of collagen fibers, and irregular arrangement of vascular smooth muscle cells (VSMCs) [2–4]. In terms of the humoral dimension, humoral markers provide valuable insights for the clinical assessment of VA due to their convenient accessibility and noninvasive (urine) or minimally invasive (blood) nature.

The vasculature plays a vital role in the proper functioning of human organs, making VA a fundamental physiological basis for age-related alterations in multiple organ systems. Moreover, VA serves as a shared etiological mechanism for various chronic diseases commonly observed in the elderly population. Of particular concern is its prominent role as a risk factor for cardio-cerebrovascular disease (CCVD). According to the seventh population census of China in 2020, the population aged 60 years and above is projected to reach 264 million, accounting for 18.7% of the total population, with the population aged 65 years and above accounting for 13.5% of the total population, further exacerbating the challenges associated with population aging [5]. Given these circumstances, the timely identification of VA and the adoption of appropriate strategies to slow and control its progression are of immense importance. These measures are critical for the prevention and management of chronic diseases in the elderly population, as well as for effectively addressing the escalating challenge of population aging [2].

On 1 July 2023, the Aging Biomarker Consortium (ABC) [4, 6–8] convened a seminar of experts in the field of VA at Tongji Hospital of Tongji Medical College, Huazhong University of Science and Technology, Wuhan, China. Through extensive literature reviews, examination of peer-reviewed research from both domestic and international scientists, utilization of evidence-based medicine, and incorporation of the unique perspectives of Chinese experts, an expert consensus on biomarkers of VA has been formed. These VA biomarkers aim to address pertinent clinical questions such as “What is the biological age of an individual’s vascular system?,” “How rapidly is the individual’s vascular system aging?,” and “How close is the individual to age-related vascular disease?”

## Methods

Literature searches were conducted for studies published before July 2023 and indexed in MEDLINE, PubMed, Cochrane Library, and other selected databases relevant to this consensus. For the specific search terms utilized, readers are referred to the online data supplement, which contains the final evidence tables summarizing the evidence used by the consensus writing group to formulate the recommendations. To initiate this process, the members of the ABC engaged in online collaboration to identify a list of key questions related to VA biomarkers. This initial compilation was based on available publications and the collective research conducted by ABC members. Subsequently, the identified VA biomarkers were thoroughly deliberated, and consensus was achieved during an expert consensus validation meeting on VA biomarkers held in Wuhan on 1 July 2023 (Wuhan 2023). All recommendations were subjected to a comprehensive review and discussion among the ABC members, allowing for diverse perspectives and considerations to be taken into consideration for this consensus.

This consensus adheres to internationally accepted conventions for expressing the level of evidence and strength of recommendations, which are detailed in Table 2 [9].

**Table 2.** Class of recommendation and level of evidence [9]

Class (strength) of recommendation	Level (quality) of evidence
Class I (strong) benefit >>> risk	Level A
Suggested phrases for writing recommendation Is recommended/is indicated Evidence and/or general agreement that a given treatment or procedure is beneficial, useful and effective	Data derived from multiple randomized clinical trials or meta analyses
Class IIa (moderate) benefit >> risk	Level B
Suggested phrases for writing recommendation Should be considered Weight of evidence/opinion is in favor of usefulness/efficacy	Data derived from a single randomized clinical trial or large nonrandomized studies
Class IIb (weak) benefit ≥ risk	Level C
Suggested phrases for writing recommendation May be considered Usefulness/efficacy is less well established by evidence/opinion	Consensus of expert opinion, and/or small studies, retrospective studies, registries
Class III (strong) risk > benefit	Note: COR and LOE are determined independently (any COR may be paired with any LOE). COR, class of recommendation; LOE, level of evidence.
Suggested phrases for writing recommendation Is not recommended Evidence/general agreement that the given treatment/procedure is not useful/effective and sometimes maybe harmful	

## Classification and clinical application of VA biomarkers

VA involves a broad spectrum of changes occurring at multiple levels, spanning from molecular and cellular alterations to organ and systemic modifications [2–4, 10, 11]. VA biomarkers, both qualitative and quantitative, should serve as reliable indicators of biological age, vascular structure, and vascular function, and can be used to determine the extent of VA and evaluate the efficacy of aging interventions. Given the gradual nature of the vascular structure and functional deterioration with age, longitudinal studies are often necessary to corroborate these changes. In such investigations, noninvasive and minimally invasive measurements, along with body fluid tests (blood, urine, etc.), are particularly favored. Furthermore, the selection of assessment methods should take into account factors such as sensitivity, convenience, cost-effectiveness, and the ability to effectively capture major changes in VA. This consensus proposes the screening of VA biomarkers from three dimensions: vascular functional characteristics, structural characteristics, and humoral markers, with the aim of enhancing both clinical practice and future research endeavors.

### Functional characteristics and assessment of VA

Functionally, VA is primarily characterized by increased stiffness and abnormal sensitivity to vasodilatory and vasoconstrictive factors, corresponding to increased vascular stiffness and endothelial dysfunction, respectively [2–4]. In addition, VA is associated with reduced transport and exchange functions of microvessels.

#### Increase in vascular stiffness

##### Pulse wave velocity

The most common clinical method for detecting vascular stiffness is measuring the pulse wave velocity (PWV). The PWV quantifies the speed at which a pressure wave travels along the arterial wall after each cardiac ejection. This measurement is obtained by assessing the time difference (_∆_*T*) and distance (_∆_*L*) between two different locations in the arterial tree using a vascular screening device (PWV = _∆_*L*/_∆_*T*). In clinical practice, the carotid-femoral PWV (cfPWV) is the golden standard for evaluating vascular stiffness. For convenience, the brachial-ankle PWV (baPWV) is often used as an alternative method [12, 13]. Both cfPWV and baPWV reflect the stiffness degree of large and medium arteries at different segments of the arterial tree and increase with age. A cfPWV study involving 146 community volunteers in the United States (96 males and 50 females) showed that from age 21 to 95, the cfPWV can increase from ~480 cm/s to ~1050 cm/s, an approximate increase of 120% [14]. Another Chinese population study involving 524 rural volunteers showed that the cfPWV can increase from ~550 cm/s at birth to approximately 990 cm/s at age 90, an approximate increase of 80% [15]. Several studies have shown that from age 20 to 70, the baPWV can increase from ~1200 cm/s to ~1500 cm/s, an approximate increase of 25% [16–18]. Moreover, increases in the PWV are closely associated with the risk of various CCVDs, chronic kidney disease, and all-cause mortality [19–22]. After adjustment for age, sex, and risk factors, each 1 m/s increase in the PWV can increase the risk of cardiovascular events and all-cause death by 14% and 15%, respectively, indicating that the PWV is a strong predictor of cardiovascular events [23]. At the same time, there is a reciprocal positive feedback effect between the PWV and hypertension, exacerbating various CCVDs caused by hypertension [24, 25].

Magnetic resonance imaging (MRI) allows the evaluation of the PWV, and phase-contrast (PC) sequences are frequently used MRI sequences for quantifying the PWV [26]. PC sequences allow for velocity encoding in one to three spatial dimensions. Four-dimensional flow (4D-FLOW) MRI is a leading-edge imaging technique that offers a more comprehensive picture of the heart and blood vessels. The fourth dimension is motion, that is, the flow dynamics during a cardiac cycle, which allows clinicians to better visualize blood flow through the cardiovascular system and potentially identify areas that require closer follow-up. In addition to providing PWV parameters, 4D-FLOW MRI can also be used to calculate parameters such as blood flow volume, flow velocity, vessel wall shear stress, pressure gradients, energy loss, and blood flow components [27]. However, the main disadvantages of 4D-FLOW MRI are the low temporal resolution, long scan times, and the need for closed source postprocessing software, which limits its widespread use in routine clinical practice [28, 29].

Numerous studies have suggested that the aortic PWV increases with age based on two-dimensional phase-contrast (2D-PC) MRI and that age affects the stiffness of different regions of the aorta to varying extents [26]. However, there is still controversy regarding the most significant region of aortic PWV increase in various study results. A recent cross-sectional study from the Multi Ethnic Study of Atherosclerosis (MESA) reported that participants aged 45–54 years had a more significant increase in the aortic arch PWV than those aged 54 years and older [30]. A study using 4D-FLOW MRI showed that the age-related differences in the PWV were greater in the proximal aortic region than in the distal descending aorta [31]. Conversely, another study using PC-MRI showed that the most significant age-related increase in the PWV was observed in the distal aorta, while the smallest increase was observed in the aortic arch, suggesting a compensatory capacity against arterial stiffening [32].

##### Cardio-ankle vascular index

The assessment of vascular stiffness can also be performed using the cardio-ankle vascular index (CAVI). In contrast to the PWV, which can be influenced by blood pressure during measurement and can provide information on the stiffness of different arterial segments by selecting specific measurement points, the CAVI serves as a blood pressure-independent index for the assessment of arterial stiffness. The CAVI is derived through calculations that incorporate electrocardiograms, phonocardiograms, ankle artery pulse waveforms, and brachial artery pulse waveforms, allowing quantification of the stiffness of the major arteries from the heart to the ankle. A higher number indicates a higher degree of artery stiffness. A CAVI of less than 8.0 is within the normal range, while the range of 8.0–9.0 is considered critical, and a CAVI greater than 9.0 indicates stiffened arteries [33].

The CAVI shows a positive correlation with age, increasing by an average of 0.5 per decade, and the average CAVI for men is ~0.2 higher than that for women across all age groups. It is a reliable indicator of the overall stiffness of the descending aorta [34]. Compared with people with a CAVI of less than 9, those with a CAVI of more than 10 have a 2.25-fold increase in the cumulative incidence of vascular diseases such as coronary heart disease and stroke [34]. Furthermore, its correlation with metabolic diseases and atherosclerosis is superior to that of the baPWV [33, 35].

##### Blood pressure indicators for vascular stiffness

The ankle-brachial index (ABI) is the ratio between the systolic pressures of the posterior or anterior tibial artery at the ankle and the brachial artery in the arm. The resting ABI of a healthy individual is typically between 0.9 and 1.3. Because pressure in the arteries of the lower limbs is usually greater than that in the upper limbs, the resting ABI of a healthy person is usually greater than 1.0. An ABI below 0.9 suggests varying degrees of narrowing or even occlusion in the peripheral arteries of the lower limbs, known as peripheral arterial disease (PAD), potentially exposing the patient to risks such as claudication, ambulation problems, and even amputation [36, 37]. The ABI is not only a method for assessing arteriosclerosis in the lower limbs and its outcomes but is also a predictive indicator for cardiovascular and cerebrovascular complications. Compared to individuals with a normal ABI, individuals with an ABI below 0.90 have significantly higher rates of cardiovascular events, cardiovascular mortality, and all-cause mortality, regardless of cardiovascular risk factors [38, 39]. In addition, calcification and hardening of the large arteries such as the aorta or narrowing of arteries such as the subclavian or carotid arteries can lead to an elevated ABI. An ABI greater than 1.4 is also an independent risk factor for cardiovascular disease [40]. Because an abnormal ABI reflects obstruction in the large and middle arteries between the ankle and brachial arteries, an abnormal ABI often interferes with the measurement of the baPWV. Therefore, the ABI is usually measured simultaneously with the baPWV in clinical practice, and together, they serve as essential indices for assessing vascular stiffness and predicting all-cause mortality in the elderly population [41, 42].

Vascular stiffness can also be reflected by parameters such as the pulse pressure (PP) and estimated pulse wave velocity (ePWV).

Pulse pressure is the difference between systolic and diastolic pressure and reflects the degree of stiffness in large arteries. The average pulse pressure in healthy adults around the age of 40 is ~40 mmHg and can gradually increase to ~60 mmHg by the age of 71–75 [43]. In addition, various pathological factors, including arteriosclerosis, can lead to an increase in pulse pressure [44]. An increase in pulse pressure is the primary pulsatile force leading to VA in middle age and beyond [45]. Compared with normal individuals, people with elevated pulse pressure have an increased risk of cardiovascular events such as coronary heart disease, heart failure, and stroke and a significantly increased all-cause mortality rate, especially in those with a pulse pressure greater than 80 mmHg [44]. The Framingham Heart Study (FHS) has shown that for every 10 mmHg increase in pulse pressure, the risk of coronary heart disease increases by an average of 23% [46], and for every 16 mmHg increase, the risk of heart failure increases by an average of 55% [47].

The ePWV is a noninvasive index calculated from age and blood pressure. The calculation formula is as follows: ePWV = 9.587 – 0.402 × age – 4.560 × 10^−3^ × age^2^ – 2.621 × 10^−5^ × age^2^ × MBP + 3.176 × 10^−3^ × age × MBP – 1.832 × 10^−2^ × MBP, where mean blood pressure (MBP) = systolic pressure + 0.4 × (diastolic pressure – systolic pressure) [48, 49], distinct from the conventional calculation of physiologic MBP. The ePWV has good consistency with cfPWV results, and the ePWV is associated with combined cardiovascular endpoint events and is independent of both Systematic Coronary Risk Evaluation (SCORE) scores and Framingham Risk Scores (FRS) [48, 49]. Vlachopoulos et al.’s secondary analysis of data from the Systolic Blood Pressure Intervention Trial (SPRINT) found that the ePWV had a predictive role for cardiovascular endpoint events in the SPRINT study population and was similarly independent of the FRS, suggesting a potentially complex relationship between the ePWV and vascular stiffness [49]. This may aid clinical cardiovascular risk assessment.

##### Ultrafast ultrasound imaging PWV

Ultrafast ultrasound imaging PWV (ufPWV) is a novel technique developed in recent years to measure the PWV based on ultrafast ultrasound imaging [50]. In this technique, the displacement trajectory of the carotid intima during the passage of the pulse wave is recorded in real-time using an ultrafast snapshot method. It directly measures the carotid ufPWV at the beginning and end of the systolic phase within one cardiac cycle. This technology is one of the most important clinical applications of ultrafast ultrasound imaging. The imaging frame rate of this technique can reach 10,000 frames per second, which is 100 times faster than the imaging speed of currently available conventional ultrasound diagnostic equipment. The ultrahigh time resolution makes it possible to measure the speed of local arterial pulse waves.

Numerous studies in China and internationally have demonstrated that carotid local elasticity measurement using the ufPWV is a reliable method for early detection and quantification of atherosclerosis and an effective risk predictor of chronic diseases such as hypertension, diabetes, and stroke [50–54]. A prospective multicenter cohort study in China first established the standardized detection method and normal reference values for the ufPWV of the carotid artery in Chinese Han adults [51]. However, the ufPWV technique also has certain limitations. Due to the large wavelength of the pulse wave and the fact that arterial plaques can affect the propagation of the pulse wave, the success rate of ufPWV measurements in carotid arteries with plaques is relatively low [50].

##### Decreased arterial strain and distensibility

Arterial strain and distensibility are important indicators of arterial stiffness. Arterial strain refers to the deformation of the elastic arterial wall due to blood flow pressure or pulsation, and the ratio of arterial strain to pulse pressure can be used to quantify arterial distensibility [55]. Axial strain is obtained by calculating the relative changes in arterial diameter or cross-sectional area during a cardiac cycle, while longitudinal strain is obtained by evaluating the relative changes in aortic length during systole and diastole. The arterial strain rate is inversely related to arterial stiffness and the PWV.

Currently, most studies assess arterial strain using cine-MRI sequences, with a few using PC-MRI or retrospective electrocardiogram-gated computed tomography (CT). PC-MRI has a lower resolution for assessing axial strain and limited accuracy in identifying aortic borders, resulting in lower repeatability compared to cine-MRI. CT is also rarely used to assess axial strain. However, assessment of longitudinal arterial strain using cine-MRI, PC-MRI, and CT shows good intra- and inter-observer agreement. These imaging modalities have shown that both axial and longitudinal arterial strain in humans are inversely related to age and show regional and sex differences. The ascending aorta has higher strain values than the descending aorta, and women tend to have greater longitudinal strain in the proximal aorta than men [26, 56–58]. However, most studies on arterial strain have focused primarily on the assessment of the aorta, and there is a lack of studies on the assessment of age-related strain in elastic arteries beyond the aorta.

#### Vascular endothelial dysfunction

Vascular endothelial cells (VECs) perceive and respond to stimuli from the blood system by synthesizing and secreting a variety of vasoactive substances, including vasodilators such as nitric oxide (NO) and prostaglandins and vasoconstrictors such as angiotensin II and endothelin-1. These substances regulate the contraction and relaxation of VSMCs, control blood pressure, and modulate vascular inflammatory responses [59–63]. Endothelial aging can lead to a series of endothelial dysfunctions, including impaired endothelium-dependent vasodilation, angiogenesis, and barrier function, which are significant risk factors for various cardiovascular and metabolic diseases, including atherosclerosis [64, 65].

The primary method used in clinical practice to assess vascular endothelial function involves the use of ultrasound technology to quantify the dilation of the brachial artery resulting from the activation of VECs and subsequent release of nitric oxide (NO) in response to shear stress. This measurement, known as flow-mediated dilation (FMD), is an indicator of overall endothelial function within the larger vessels of the brachial artery [66, 67]. FMD declines with age and is an independent prognosticator of future cardiovascular events and outcomes associated with cardiovascular disease (CVD) [66, 68, 69].

Microvascular endothelial function can be assessed by determining the reactive hyperemia index (RHI), which is obtained by assessing the peripheral resistance of the fingertip microvasculature using a peripheral arterial tonometry system [66, 70]. The RHI can be measured using the Endo-PAT2000® device (Itamar Medical Ltd., Israel), with 1.67 serving as the cutoff value. An RHI of 1.67 or above is considered normal, while an RHI <1.67 indicates endothelial dysfunction [71]. The RHI is associated with the burden of atherosclerosis [72, 73] and can also independently predict future cardiovascular risk [69]. However, it does not reflect the age-related decrease in large vessel endothelial function assessed by FMD, suggesting that the RHI and FMD may reflect different aspects of vascular endothelial function [74, 75]. Recent studies have shown that the RHI is associated with glucose and lipid metabolism [71, 76]. Metformin intake can significantly improve the RHI value in polycystic ovary syndrome patients with endothelial dysfunction (RHI = 1.3 ± 0.3) [76], suggesting that the RHI may reflect microvascular endothelial function related to glucose and lipid metabolism. The vascular function reflected by the RHI needs to be validated by further clinical cohort studies and basic mechanistic research.

#### Reduced transport and exchange function and microcirculation disorders

The microcirculation, composed of microvessels, is the site where the exchange of gases and substances takes place between vessels and various tissues and organs throughout the body. Aging is associated with changes in the microvascular diameter, structure, and distribution density, which together lead to changes in the microcirculation. Recent studies have shown that the distribution density of microvessels decreases significantly with aging, reducing the surface area available for substance exchange between blood and tissues and severely impacting the efficiency of substance exchange [77]. Clinically, noninvasive detection techniques such as laser speckle contrast imaging (LSCI) and laser Doppler blood flow imaging [78–80] can be used to measure blood flow and microvascular density in the skin microcirculation in real time to reveal changes in vascular transport and exchange function during aging. This may serve as a functional marker for the assessment of VA.

Coronary microvascular disease (CMVD) is characterized by structural and functional abnormalities of the coronary microvasculature leading to local myocardial ischemia. Age is a major risk factor for CMVD. Clinically, coronary flow reserve (CFR) and the index of microcirculatory resistance (IMR) are used to quantify CMVD. However, both the CFR and IMR are obtained by invasive methods, making them unsuitable for early screening. Myocardial perfusion imaging (MPI) [81] can accurately assess endocardial and epicardial myocardial microcirculation, coronary resistance and diastolic filling time, reflecting myocardial ischemia and coronary microvascular obstruction. Thus, the MPI is an important noninvasive imaging modality for the diagnosis and evaluation of myocardial microcirculatory disorders. However, the MPI has limitations such as long scan times, susceptibility to respiratory and motion artifacts, the need for multiple breath holds, and the risk of inducing myocardial ischemia during stress perfusion imaging, which limits its clinical implementation.

Changes in the diameter of intracerebral microvessels show a positive correlation with the ratio of cerebral blood flow (CBF) to cerebral blood volume (CBV) [82]. Recently, a retrospective cohort study based on 4D-FLOW showed that in a cognitively normal population aged 45–93 years, cerebral blood flow as measured by 4D-FLOW decreased by 4 mL/min/year, while the pulsatility index in all cerebral vascular segments increased by an average of 0.01 arbitrary units (au) per year. The decrease in total cerebral blood flow measured by 4D-FLOW, the decrease in blood flow in specific vascular segments, and the increase in the pulsatility index shows a strong correlation with age [83].

The cerebrovascular reserve (CVR) reflects the ability of small cerebral arteries to maintain cerebral blood flow stability or to regulate cerebral blood flow to meet the demands of brain function by dilation or constriction. A decrease in CVR has been shown to reflect microcirculatory disturbances earlier than a decrease in CBF [84]. Imaging assessment of the CVR includes positron emission tomography (PET) and single-photon emission computed tomography, among others. PET, with a spatial resolution of 4–6 mm and high precision, is considered the current gold standard for CVR detection. However, it is not widely used due to its complex equipment, high cost, and radioactivity. Arterial spin labeling (ASL) imaging can detect changes in blood flow perfusion caused by various vascular tensions without the need for contrast agent injection and has the potential to evaluate the CVR [85]. The diffusion-prepared pseudocontinuous arterial spin labeling (DP-pCASL) imaging derived from ASL can also be used to assess abnormalities in blood–brain barrier permeability and water channel function [86].

CVR reflects the body’s ability to adapt to changes in cerebral oxygen demand by regulating CBF. When the CVR is impaired, metabolic reserves are immediately mobilized to increase the oxygen uptake capacity of brain tissue. Blood oxygenation level-dependent functional MRI (BOLD-fMRI), which depends on blood oxygen levels, is commonly used to assess metabolic reserve function and can be used to calculate the oxygen extraction fraction (OEF) [87]. However, BOLD-fMRI essentially reflects blood oxygen saturation, and BOLD-fMRI is affected by the functional state of the vasculature at the time of examination, with a high degree of variability, many interfering factors, and low temporal resolution. Quantitative susceptibility mapping (QSM) is a new technique for measuring the OEF. QSM uses gradient-echo phase maps to reflect the distribution of magnetically sensitive substances and calculates their susceptibility values, which reflect changes in the content of deoxygenated hemoglobin in cerebral veins, thereby quantifying the OEF [88]. The CVR and OEF are potential imaging techniques for assessing age-related microcirculatory changes.

#### Recommendations

- (1) Increased vascular stiffness and endothelial dysfunction are functional markers of VA. In clinical practice, the stiffness of the vessels can be assessed by combining the baPWV with the ABI, and endothelial dilation function can be assessed by FMD (Level A evidence, Class I recommendation).
- (2) The CAVI, ufPWV, PP, and ePWV reflect arterial stiffness in different segments and areas and could be considered functional markers of VA (Level B evidence, Class IIa recommendation).
- (3) The RHI is an indicator of microvascular endothelial function and can be considered a functional marker of VA, but it needs to be verified in future cohort studies (Level B evidence, Class IIa recommendation).
- (4) Reduced arterial strain can serve as a marker of vascular functional aging, and arterial strain can be assessed using MRI and CT (Level B evidence, Class IIa recommendation).
- (5) LSCI and laser Doppler can noninvasively detect subcutaneous microcirculation and capillary density and may be considered functional markers of VA. However, they need to be verified in future cohort studies (Level B evidence, Class IIb recommendation).
- VA is associated with microcirculatory disturbances related to changes in blood flow and blood volume. Observations of reduced cerebral blood flow and increased pulsatility using 4D-FLOW may serve as markers of vascular functional aging (Level B evidence, Class IIb recommendation).

### Structural characteristics and assessment of VA

Aging vessels undergo microscopic and macroscopic morphological changes, leading to a decline in their function and subsequent hemodynamic imbalance. Structural assessment of VA involves the analyses of morphological changes in blood vessels at microscopic and macroscopic levels. Imaging techniques such as vascular ultrasound, computed tomography (CT), and magnetic resonance imaging (MRI) can be used to analyze the morphological and functional changes in aging blood vessels at multiple scales. These imaging techniques provide visual, quantitative, and imaging biomarkers to describe the process of VA and its progression to vascular diseases.

#### Changes in geometric characteristics

The integrity of the vascular structure is essential for the maintenance of vascular function. Elastic arteries serve two main functions, namely, conduit and cushion, designed to transport blood to the periphery and to buffer the pulsatile stresses of ventricular contraction. However, with age, arteries undergo structural changes, including arterial wall thickening, luminal dilation, and increased vessel length and tortuosity. Noninvasive methods such as vascular ultrasound, CT angiography (CTA), and MR angiography (MRA) are reliable for assessing the geometric features of the VA.

An essential structural characteristic of VA is the increase in vascular wall thickness, which can be assessed noninvasively using ultrasound to measure the carotid intima-media thickness (cIMT) [89]. cIMT refers to the distance between the intimal surface and the outer surface of the arterial wall in the carotid artery, measured using a high-frequency B-mode ultrasound probe. The measurement site is typically 1.0–1.5 cm proximal to the far wall of the carotid bifurcation. In the presence of plaque, the measurement is performed 1.0–1.5 cm proximal to the lesion site [2, 89]. The linear increase in cIMT with age is evident in healthy individuals aged 21–105 years, and it can be approximated by the formula cIMT = (0.009 × age) + 0.116 (*r* = 0.83). Notably, cIMT remains a better predictor of chronological age than plaque burden and the PWV [90]. Elevated cIMT serves as an independent risk factor for acute cardiovascular events [91], and in individuals with atherosclerosis risk factors, VA is accelerated, leading to an increased rate of cIMT growth [92–94]. Currently, increased cIMT is considered a hallmark structural change of VA and is often used as a secondary endpoint in clinical trials. Early detection of elevated cIMT is critical for slowing VA and preventing and intervening in the subclinical development of atherosclerotic vascular disease [6]. However, it should be noted that ultrasound measurements of vessel wall thickness are mainly limited to superficial arteries, such as the carotid and femoral arteries, and deep vessels are difficult to access.

CTA and MRA are advanced imaging techniques known for their high accuracy and reproducibility in quantitatively assessing vascular diameter, cross-sectional area, length, and tortuosity. These techniques are widely used for geometric measurements of large and medium-sized arteries and are essential for the diagnosis and evaluation of vascular diseases associated with aging, including abdominal aortic aneurysm and aortic dissection. The MESA study demonstrated that the mean diameter of the aorta increases by 1.1 mm per decade in a multiethnic population aged 45–85 years, with men showing a greater increase than women [95]. Additionally, numerous studies have shown that aortic length and curvature also increase with age, although they are influenced by various factors, such as height, body size, posture, and spinal curvature. This leads to high individual heterogeneity, suggesting that these measures are sensitive indicators of VA but may have limited specificity [26].

In clinical practice, high-resolution digital subtraction angiography (DSA) is often used to assess the geometric morphology of small blood vessels, particularly to visualize collateral vessels supplying the basal ganglia and adjacent tissues in the brain. However, it is important to recognize that DSA is an invasive technique that carries the potential risk of exposure to X-ray radiation. As an alternative approach, 7.0 T ultrahigh-field time-of-flight MRA (TOF-MRA) offers a noninvasive means of visualizing intracranial collateral vessels, providing high-resolution imaging capabilities and allowing quantification of the collateral length, tortuosity and number of branches [96]. However, it should be noted that the clinical application of 7.0 T MR is limited by a nonuniform magnetic field, susceptibility artifacts, and long scanning time.

Recently, vessel wall MRI (VWMRI) has made significant progress. Zhang et al. [97] developed a clinically feasible *in vivo* VWMRI method at 3.0 T for imaging the basilar artery and successfully extracted the structural skeleton of the collateral vessels. This study demonstrated that 3.0 T VWMRI can clearly visualize the main trunk and proximal branches of the collateral vessels in healthy volunteers, with no significant difference compared to 7.0 T TOF-MRA. The use of VWMRI provides a new method for the quantitative assessment of vessel structure from the macroscopic to the mesoscopic level, which may provide future insights into the assessment of collateral vessel aging.

VWMRI is advantageous for visualizing deep vascular structures. In the MESA study, spin-echo T1-weighted black-blood MRI sequences were used to reveal a trend of increased average wall thickness in the descending aorta with advancing age. Specifically, in the baseline population aged 45–54 years, wall thickening in the descending aorta was more pronounced and reached a stable period with increasing age [98]. However, MRI does have limitations in spatial resolution for measuring aortic wall thickness. The in-plane spatial resolution ranges from 0.7 to 1 mm, which is approximately half the thickness of the aortic wall. To save scan time, the slice thickness is usually greater than 5 mm, which impacts the accuracy of vascular wall thickness measurements due to the partial volume effect. To overcome this problem, recent developments have led to the creation of three-dimensional, high-resolution (0.6 mm isotropic) VWMRI methods capable of clearly displaying the intimal and adventitial walls of arteries and indicating positive or negative remodeling of the vessels. These methods demonstrate high reproducibility and good inter- and intra-observer agreement and are mainly used for quantitatively measuring the wall thickness of the intracranial arteries and carotid arteries [99]. Notably, Yang et al. [100] proposed a novel VWMRI method with high spatial resolution and large coverage, which revealed irregular wall thickening of the intracranial arteries in elderly patients with coexisting intracranial atherosclerosis [101]. Currently, VWMRI is recommended for measuring the wall thickness of the intracranial arteries, carotid arteries, and the aorta. VWMRI has also been used to measure arterial wall thickness in the lower limbs [102], but the current study did not differentiate between age groups.

#### Vascular calcification

Vascular calcification refers to the abnormal deposition of calcium salts in blood vessels, which is a characteristic of VA. During the aging process, VSMCs undergo replicative senescence, and age-related chronic kidney disease-induced hyperphosphatemia can induce the osteogenic transformation of VSMCs through various mechanisms. This leads to the deposition and formation of calcified plaques on the elastic fiber membrane in the media, resulting in the disruption and remodeling of the extracellular matrix (ECM). Consequently, vascular elasticity decreases, stiffness increases, and vascular medial calcification and aging are promoted [103]. In addition, in patients with atherosclerosis, various factors, such as plaque progression, lipid accumulation, oxidative stress, and inflammatory necrosis, stimulate transformation and functional changes in vascular cells. This process promotes the deposition of calcium-phosphate crystals in the lipid necrotic core of atherosclerotic plaques, giving rise to the development of calcified plaques and, in severe cases, ossification. This type of calcification is known as intimal atherosclerotic calcification [104]. Vascular calcification is prevalent among individuals aged 70 and older, with 93% of men and 75% of women exhibiting varying degrees of vascular calcification. Vascular calcification can manifest in all vascular beds, and the level of calcification is consistently associated across these beds [105].

CT is sensitive enough to detect calcification and is the preferred imaging method for assessing vascular calcification. It can also be used to differentiate between medial and intimal calcification, assess plaque burden, and predict CVD risk and outcomes [106]. Currently, clinical guidelines recommend using CT to determine the coronary artery calcium score (CACS), which classifies calcification into categories of no calcification (CACS = 0), mild calcification (CACS = 1–99), moderate calcification (CACS = 100–400), and severe calcification (CACS > 400) based on the CACS [107]. The CACS is highly correlated with age and is the strongest predictor of atherosclerosis-related heart disease. This method is widely used in clinical practice for cardiovascular risk assessment. Recently, there have been studies using CACS to classify vascular age for risk stratification of atherosclerotic CVD [108]. Additionally, there are studies that quantify the degree of calcification in the aorta and lower limb arteries using CT or CTA [109], but a complete evaluation system has not been established, and large-scale population cohort studies are lacking.

It is widely believed that MRI cannot effectively assess vascular calcification due to the lack of protons in calcified plaques. However, with advances in MRI technology, novel sequences such as QSM and simultaneous noncontrast angiography and intraplaque hemorrhage (SNAP) have emerged to enable the identification and assessment of calcification in the carotid arteries. QSM uses the phase information of MRI to capture local magnetic field variations in tissue. By exploiting the relationship between the magnetic field map and magnetic susceptibility, QSM quantitatively measures tissue magnetization characteristics. This technique is effective in analyzing tissue parameters such as iron content, calcification, and blood oxygen saturation. Recent studies have shown that QSM sequences can distinguish vascular calcification from intraplaque hemorrhage components, as calcification exhibits diamagnetic properties, whereas focal hemorrhage or hemosiderin exhibits paramagnetic properties [110, 111]. SNAP is a high-resolution vascular wall imaging technique derived from phase-sensitive inversion recovery sequences [112]. This method allows the simultaneous acquisition of multiple contrast images, improving the differentiation between plaque surface calcification and the blood pool signal. Clinically, SNAP can be used to identify both calcification and intraplaque hemorrhage [113]. Despite these recent advances, there are currently no studies using MRI to assess age-related vascular calcification. However, these emerging MRI sequences hold promise for the future assessment and understanding of vascular calcification in the context of VA.

#### Abnormalities in the microvascular distribution and structure

Emerging evidence suggests that the microvascular system not only facilitates substance exchange between blood vessels and tissues but also serves as a dependable indicator of the overall health of the circulatory system and the brain. Subtle changes in the vasculature often first manifest themselves in the smallest blood vessels.

Retinal microvessels, some of the smallest blood vessels in the body, can be detected by fundoscopic examination (FE) using an ophthalmoscope and the naked eye [114]. FE is recommended for the prevention and diagnosis of retinal lesions and visual impairment in diabetic patients [115, 116]. In addition, FE noninvasively identifies ocular microvascular abnormalities such as arterial and venous occlusive disease, arteriovenous malformations, and embolic events, thereby helping to prevent and manage ocular and systemic complications associated with hypertension [117]. As a result, FE can be used to assess microvascular complications closely related to increased vascular stiffness and to improve prognosis and risk reclassification in VA and related diseases [118, 119].

The development of deep learning models by various research teams in China and abroad has demonstrated high accuracy in capturing signs of aging in retinal images, enabling the prediction of biological age. These models have established retinal age or retinal aging clocks [120, 121]. Research has shown that for every 1-year increase in the retinal age gap (the difference between the retinal age and chronological age), the risk of death increases by 2% [121]. Notably, retinal age remains an independent risk factor for all-cause mortality, with a hazard ratio of 1.026, even after adjustment for phenotypic age [120]. These findings highlight the potential of retinal imaging in assessing VA and its impact on general health and mortality risk.

Optical coherence tomography (OCT) is a remarkable noninvasive three-dimensional optical imaging technique capable of high-resolution imaging of living microvessels without the need for contrast agents. It offers numerous advantages, including a short scanning time, fast processing speed, good imaging depth, and immunity to the effects of blood flow direction. Winkelmann et al. [122] introduced a novel spectral contrast OCT imaging method that allows the visualization of capillaries as small as 4 μm in diameter. OCT can also quantify the capillary density, providing an indirect measure of the capillary regenerative capacity. A study using OCT confirmed a significant difference in capillary density between young and aged mice, with aged mice showing a 15% reduction in capillary density compared to that of their younger counterparts. Furthermore, advanced OCT algorithms accurately quantified capillary blood flow velocity, revealing a 21% reduction in the mean capillary blood flow velocity in aged mice compared to young mice [102]. Thus, OCT is emerging as a promising imaging modality for assessing microvascular changes.

Numerous studies have shown a decrease in vascular density during the natural aging process in both animals and humans. In a cohort study of 100 healthy volunteers, reductions in vessel density were observed in the anatomical regions of the middle cerebral arteries, anterior cerebral arteries, and posterior cerebral arteries as visualized by MRA. Specifically, participants aged 60 years and older had reduced vessel counts in the anatomical regions of the middle cerebral arteries, with a significantly lower number of posterior circulation vessels compared to those of other age groups [123]. In addition, the relationship between the cerebral medullary veins and age-related brain atrophy is strong, making it a key target for the assessment of microvascular structural abnormalities in aging. Magnetic resonance imaging (MRI) with susceptibility-weighted imaging (SWI) allows visualization of the morphology and number of intracranial small veins, providing insight into pathological changes, including microhemorrhages in the brain. A cross-sectional study [124] found a negative correlation between SWI-measured medullary vein density and the score for age-related dementia, suggesting that medullary vein density observed on SWI in the brain’s white matter serves as a sensitive marker for different stages of cognitive and functional status in cerebral oxygen metabolism and neurodegenerative diseases.

#### Recommendations

(1) Increased cIMT measured by ultrasound is a marker of structural change in VA (Level A evidence, Class I recommendation).
(2) FE is recommended for assessing retinal microvascular aging (Level A evidence, Class I recommendation).
(3) An increased CACS indicates arterial calcification and can differentiate between medial and intimal calcification, assesses atherosclerotic plaque burden, and is a structural marker of VA (Level A evidence, Class IIa recommendation).
(4) The use of CTA and MRA can noninvasively evaluate the geometric configuration changes of aging vessels and observe the changes in vessel diameter, lumen cross-sectional area, length, and tortuosity, which can be used as structural markers of VA (Level B evidence, Class IIa recommendation).
(5) The use of OCT can noninvasively assess the capillary density, providing an indirect measure of the capillary regenerative capacity, and can be used as a structural marker of VA (Level B evidence, Class IIa recommendation).
(6) MRA and SWI can detect decreases in cerebral vascular density in the aging population and can be used as structural markers of VA (Level B evidence, Class IIa recommendation).
(7) Increased vascular wall thickness based on VWMRI measurement may suggest VA (Level B evidence, Class IIb recommendation).

### Humoral biomarkers

Humoral markers are highly suited for the clinical assessment of VA due to their convenient accessibility and noninvasive (urine) or minimally invasive (blood) nature. In the aging population, early detection of fluid markers can effectively identify individuals with increased susceptibility to vascular damage, thereby reducing age-related CVD and its associated socioeconomic burden. In routine clinical settings, venous blood is typically obtained, and the constituents present in venous blood include the byproducts of the interaction between blood and tissues, including the secretory and metabolic products of tissues and organs. However, these constituents do not fully represent the various nutrients delivered by the blood to the tissues and organs, nor do they fully reflect the secretion and metabolism of the vascular tissues themselves. Nevertheless, the components present in the blood can directly affect vascular tissues, particularly the *intima*, and thereby influence the function and structure of vascular tissues. As a result, changes in blood markers can serve as indicators of systemic inflammation, metabolism, and aging and can be used to assess and predict VA and VA-related diseases.

### Plasma inflammatory factors

VA is closely associated with the release of inflammatory mediators. The senescence of vascular cells is accompanied by an increase in the inflammation-associated secretory phenotype (SASP) [59, 125]. At the same time, aging triggers a series of changes in the immune system that promote the production of various inflammatory factors. Together, these changes contribute to an increase in multiple inflammatory factors in the bloodstream with age, thereby promoting the progression of VA-related diseases [126, 127]. The levels of many inflammatory factors, including interleukin (IL)-6, IL-1, IL-1 receptor antagonist (IL-1Ra), IL-8, interferon γ (IFNγ), tumor necrosis factor alpha (TNFα), high-sensitivity C-reactive protein (hs-CRP), vascular endothelial growth factor (VEGF), oxidized low-density lipoprotein (ox-LDL), and reactive oxygen species (ROS), have been shown to increase in the plasma with age [6, 128–130]. Furthermore, a positive correlation was observed between the elevated red blood cell distribution width (RDW) and systemic levels of inflammatory factors, such as hs-CRP, IL-6, TNFα, matrix metallopeptidase 2 (MMP-2), and MMP-9. The inclusion of the RDW in conjunction with these inflammatory markers provides an improved means of assessing VA and aortic pathology [128]. However, due to their nonspecific nature, their susceptibility to inflammatory diseases and various CVDs, and their tendency to primarily reflect the current level of inflammation in the organism, these markers are not considered suitable for stand-alone use as indicators of VA.

#### Other plasma proteins

##### Plasma insulin-like growth factor 1

Insulin-like growth factor 1 (IGF-1) is widely expressed in various cell types and functions as both an endocrine and an autocrine/paracrine growth factor. Its presence in the circulation is maintained at elevated levels [131]. Specifically, plasma IGF-1 has been observed to have beneficial effects in slowing VA, possibly through mechanisms involving the reduction of oxidative stress, apoptosis, proinflammatory signaling, and endothelial dysfunction [132]. Numerous studies have reported a decrease in plasma IGF-1 protein concentrations with advancing age [133–135]. In a single-center cross-sectional study, plasma IGF-1 levels were found to decrease significantly with age. Specifically, the levels decreased from 175 ng/mL in youth to 125 ng/mL in elderly individuals [134, 136]. This decline in plasma IGF-1 levels is accompanied by a simultaneous reduction in cerebral blood flow during the aging process. Together, these factors contribute to neurovascular aging, impaired cerebral blood flow, and age-related cognitive decline [134, 136]. In addition, decreased plasma IGF-1 in the elderly is positively correlated with increased mortality rates [137] and negatively correlated with cIMT [138], suggesting that a decrease in plasma IGF-1 concentration may be a marker of VA and a risk factor for CVD.

##### Plasma fibroblast growth factor 21

Circulating fibroblast growth factor 21 (FGF21) is mainly expressed and secreted by the liver [139]. Plasma FGF21 concentrations have been shown to correlate positively with age [140, 141] and can be modulated by a variety of lifestyle factors [41]. The median fasting plasma FGF21 concentration increased from 113 pg/mL to 210 pg/mL among healthy Chinese individuals aged 20 to 70, and aging was identified as an independent factor contributing to this increase, with 90% of individuals aged 60–70 having plasma FGF21 concentrations below 400 pg/mL [141]. Elevated plasma FGF21 levels have been strongly associated with an increased risk of CVD, potentially through a stress response triggered by inflammation and abnormalities in glucolipid metabolism [142]. Furthermore, critical values of plasma FGF21 (ranging from 123.0 to 321.5 pg/mL) have been reported to be independent risk factors for predicting cardiovascular risk [142–144].

##### Plasma Fibulin-1

Arterial stiffening is recognized as an independent risk factor for CVD, with remodeling of the ECM of VSMCs playing a key role in this process [145]. In plasma, alterations in ECM proteins can be observed. Fibulin-1 is an important ECM protein that interacts with elastin and microfibrils and together contributes to the stabilization of elastic fibers in the vessel wall [146]. Plasma Fibulin-1 levels were elevated in individuals with increased arterial stiffness, and this elevation was positively associated with the increase in baPWV. After adjustment for confounders, including age and blood pressure, plasma Fibulin-1 levels differed significantly between the high baPWV group (2,376 ± 147.7 cm/s) and the normal baPWV group (1,171 ± 147.7 cm/s), measuring 12.69 ± 0.89 µg/mL and 9.84 ± 0.71 µg/mL, respectively (*P* < 0.05), suggesting that plasma Fibulin-1 may serve as a potential indicator of arterial stiffness [145].

##### Plasma ancient retrovirus envelope protein

Recent research has demonstrated that human endogenous retrovirus K (HERVK), a dormant element within the human genome, can be unlocked and transcribed with the loss of perinuclear heterochromatin and loosening of chromatin structure during aging. This activation leads to the production of retrovirus-like particles in tissue cells that contribute to various forms of cellular senescence and tissue aging [147]. In addition, a significant twofold increase in the concentration of the HERVK envelope protein (HERVK-Env) is observed in the serum of old individuals compared to young individuals, suggesting that the resurrection of HERVK may be a marker and driver of cellular senescence and tissue aging [147].

#### Cells and vesicles in the circulation

##### Circulating CD8^+^CD28^−^ T lymphocytes

T lymphocytes are found in high numbers in vascular tissue [148] and in the bloodstream. Aging leads to the exhaustion and loss of CD28, a costimulatory molecule found on the surface of T cells, particularly cytotoxic T lymphocytes [149]. This phenomenon is clinically manifested by a significant increase in the proportion of circulating CD8^+^CD28^−^ T-cell subsets with age [150]. In healthy individuals, the CD8^+^CD28^−^ T-cell subset in the bloodstream gradually increases from 0% at birth to ~60% of CD8^+^ T cells by the age of 80 [149], a change that can be readily detected by flow cytometry in the clinic. Compared to CD28^+^ T cells, CD28^-^ T cells have reduced proliferative capacity and increased expression and secretion of proinflammatory cytokines and play an important role in various age-related CVDs [150, 151]. Therefore, the detection of CD8^+^CD28^-^ T lymphocytes in the blood may also serve as an assessment of vascular senescence and a predictor of cardiovascular risk.

##### Circulating endothelial progenitor cells

Circulating endothelial progenitor cells (EPCs) are bone marrow-derived endothelial progenitor cells found in the peripheral blood. There is increasing support for their important role in maintaining the functional integrity of the vascular endothelium and their specific mode of action to promote the regeneration and repair of damaged endothelium by replacing dysfunctional endothelial cells [152–154]. In healthy men, the levels of circulating EPCs showed a significantly negative correlation with the Framingham risk score [155, 156]. Indeed, a decrease in circulating EPCs leads to endothelial dysfunction and impaired vascular elasticity [157]. Consequently, changes in EPC levels may serve as a biomarker for cumulative cardiovascular risk. EPCs are present at very low levels in the bloodstream, with mature CD34^+^CD144^+^ EPCs representing ~0.06% of peripheral blood mononuclear cells in adults (45.9 ± 2.1 years) [158] and even less in elderly individuals, making the detection of EPCs alongside the abundant T cells, B cells and NK cells in the blood by flow cytometry challenging and thus limiting the clinical implementation of EPC detection.

##### Circulating endothelial microparticles

Depending on their size, mode of production and release, extracellular vesicles are classified as exosomes, microparticles, and apoptotic bodies. The heterogeneity of plasma microsomes is mainly because they originate from different cells, including erythrocytes, leukocytes, platelets, and endothelial cells. These microparticles are rich in proteins, DNA, RNA, miRNA, and lipids. In most cells cultured in vitro, the levels of microparticles have been found to increase more than 10-fold during replicative senescence and damage-induced senescence [159].

Circulating endothelial microparticles (EMPs), which have diameters ranging from 0.1–1.0 μm, have been reported to have a good positive correlation with the baPWV (*r* = 0.371, *P* = 0.008) and therefore can be an important indicator of endothelial damage and increased vascular stiffness [160]. Compared to young adults (26 ± 3 years), healthy older adults (60 ± 6 years) showed a remarkable increase of 30%–50% in the plasma concentrations of CD31^+^CD41^−^, CD144^+^, and CD62e^+^ EMPs compared to their younger counterparts (26 ± 3 years). Furthermore, certain EMPs showed significant positive correlations with systolic blood pressure and PWV, while all three EMPs showed significant negative correlations with FMD [161]. In addition, circulating CD31^+^CD41^−^ and CD144^+^ EMPs have been reported to exhibit varying degrees of association with hypertension, hyperlipidemia, and metabolic syndrome [162]. Therefore, EMPs can be considered indicators of vascular damage and metabolic disorders associated with the aging process. Due to the small size of EMPs, their detection requires certain advanced technological capabilities. In addition, different research teams have defined EMPs based on different surface markers, resulting in a heterogeneous definition of EMPs. Together, these factors have limited the widespread adoption and implementation of EMP detection in the clinic.

#### Other functional markers

##### DNA methylation

Altered DNA methylation has been detected in the blood of aging individuals [163], and the presence of reduced levels of DNA methylation in the blood has been associated with the progression of atherosclerosis and increased susceptibility to cardiovascular complications [164]. In particular, abnormal changes in DNA methylation of gene-specific promoters in monocytes/macrophages in atherosclerosis patients may serve as a diagnostic indicator with significant clinical implications [165]. In a study carried out with 742 elderly men, a significant decrease in the level of DNA methylation of long interspersed element 1 (LINE-1) was observed in their blood, which was negatively correlated with increased levels of vascular cell adhesion molecule-1 (VCAM-1) in their blood [164]. In addition, plasma samples from patients with neurodegenerative diseases and senile cerebrovascular disease showed significant reductions in both global methylation and hydroxymethylation levels, as well as the mRNA levels of DNA methyltransferase 3 alpha (DNMT3α) [166]. Therefore, the reduction in total DNA methylation levels in the blood may potentially act as an indicator of VA.

In addition, DNA methylation age (DNAmAge), commonly referred to as the “epigenetic clock,” provides a comprehensive understanding of the entire biological progression from infancy to old age, allowing a more accurate assessment of the biological age (BA) of any given tissue throughout an individual’s lifespan [167–169]. Different epigenetic clocks come from different DNAmAge assessment strategies, such as Horvath’s clock, which monitors CpG methylation changes at 353 genomic loci in different tissues and cells in the human body over time [170]; Hannum’s clock, which assesses the methylation changes at 71 age-related CpG loci in blood samples only [171]; or Levine’s clock, which incorporates 10 clinical indicators (chronological age, albumin, creatinine, glucose, CRP, etc.) alongside methylation changes at 513 CpG sites in the blood for predicting phenotypic age (phenotypic age) [172]. All three DNAmAges could accurately capture the biological age of whole blood across the lifespan [167], thereby influencing and reflecting VA. Currently, these methylation clocks are mainly based on foreign population samples, which require further validation for their applicability in assessing BA and VA in Chinese individuals. It would also be necessary to establish native DNAmAge using Chinese samples.

DNAmAge can serve as a valuable VA biomarker. However, the widespread adoption of DNAmAge assessment in clinical practice is hampered by its high technical requirements and associated costs.

##### Glycosylation of circulating immunoglobulin G

Modification of immunoglobulin G (IgG) by glycosylation (specifically N-glycosylation) has been observed to affect its function and systemic inflammatory state, with most glycosylation patterns found to be closely associated with the aging process [173]. The glycosylation patterns at the asparagine 197 (Asn197) site of the heavy chain of plasma IgG were investigated using ultra-performance liquid chromatography (UPLC), and the analysis revealed the presence of 24 peaks, each corresponding to a different glycosylation pattern at this particular site. The combined analysis of three glycosylation peaks (GP6, GP14, and GP15) accounted for 58% of the variation between the chronological age of an individual and their biological age [173]. Due to the technical requirements and associated costs, it is difficult to promote this technique clinically.

##### Blood exosomal noncoding RNAs

Noncoding RNAs (ncRNAs), including microRNAs (miRNAs) and long ncRNAs (lncRNAs), play an important role in VA [174]. Dyslipidemia, hyperglycemia, and hypertension all lead to abnormally elevated miR-34a expression in vascular cells, which contributes to inflammation and VA by affecting VEC and VSMC function [175]. Significantly increased levels of miR-34a have been detected in the blood of aged patients and those with a variety of age-related diseases [176–179], suggesting that blood levels of miR-34a may also serve as a marker of aging and as a VA biomarker. Exosomal miRNAs are also potential biological markers of vascular senescence [180]. Exosomes are involved in regulating cellular physiological functions by transmitting signals to neighboring or distant cells in an autocrine or paracrine manner [180].

Regulation of VA through the modulation of VECs, VSMCs, ECM remodeling, and inflammatory factor secretion has been observed in exosome-derived miRNAs present in blood and vascular tissues [180]. For example, an association has been found between elevated plasma levels of exosomal miR-501-3p in older individuals and increased vascular stiffness [181]. Plasma exosomal miR-34a levels have also been reported to increase with aging and age-related diseases [178, 182]. However, given the short half-life of miRNAs *in vivo* and their susceptibility to various diseases and environmental factors, further validation is required to determine the suitability of miRNAs and exosomal miRNAs as viable VA biomarkers for generalized clinical application.

##### Circulating metabolites

Human metabolites are involved in a variety of physiological processes and include a diverse array of organic and inorganic macromolecules and their derivatives, such as amino acids, nucleic acids, carbohydrates, fatty acids, functional nutrients (e.g., vitamins and cofactors), and compounds such as sex hormones, drug intermediates, and toxins. The Human Metabolome Database (HMDB) has annotated over 200,000 metabolites that have the potential to be present in humans, encompassing both endogenous and exogenous molecules [183]. For the qualitative and quantitative analysis of metabolites, two commonly employed techniques are high-performance liquid chromatography–triple quadrupole mass spectrometry (LC–MS/MS) and nuclear magnetic resonance (NMR).

Blood contains a large number of metabolites that are either produced internally or taken up from the environment. These metabolites are subject to many influences, including hereditary traits, the gut microbiome and lifestyle choices such as smoking and diet. Nevertheless, the primary factors responsible for the majority of these metabolites remain unidentified [184]. Among these factors, diet and the gut microbiome have emerged as the most influential determinants in detecting and predicting the presence of circulating metabolites [184]. In particular, certain noteworthy metabolites may provide valuable insights into the likelihood of developing several prevalent diseases, such as CVD, type 2 diabetes, and dementia [185, 186].

Human aging is associated with a diverse range of physiological and pathological alterations, including changes in lipids and lipoproteins, amino acids, steroid hormones, gut microbiome, diet, inflammation, oxidative stress, and declining liver and kidney function, all of which result in changes in the fractions and concentrations of metabolites present in blood and urine [183]. For instance, numerous studies have demonstrated a correlation between aging and a decline in blood tryptophan concentrations, as well as an elevation in tyrosine concentrations [187–190], while a significant increase in plasma L-methionine levels was observed in another model of rapid aging [191]. Aging is frequently associated with elevated levels of blood cholesterol [189], polyunsaturated fatty acids (PUFAs) [192], the choline and betaine metabolite trimethylamine N-oxide (TMAO) [193, 194], and sphingolipids such as ceramide [195–197]. These molecules not only pose as risk factors for CVD but also hold potential as VA biomarkers. In addition, aging often coincides with reduced levels of certain metabolites in the blood, such as uridine, a metabolite derived from glutamine/vitamin B13. Not only does uridine show a significant reduction in blood levels in older individuals, but it has also been shown to have a variety of preventive and retarding effects on aging, including increasing stem cell viability, facilitating tissue repair and regeneration, improving cognitive function, preventing dementia, and improving sleep quality [198].

##### Metabolites in urine

The aging process is closely linked to metabolism and oxidative stress. As urine serves as a repository of body changes, it represents a valuable resource for identifying aging biomarkers. Studies using proteomic and metabolomic analyses of urine samples from aging rats and humans have shown that the proteins and metabolites that show age-related changes are primarily associated with renal aging [199–201].

A 2012 study using LC-MS/MS examined the blood and urinary levels of oxidation products of DNA and RNA in rhesus monkeys (*Macaca mulatta*), demonstrating the presence of 8-oxo-7,8-dihydrodeoxyguanosine (8-oxo-dGsn), an oxidative product derived from DNA, and 8-oxo-7,8-dihydroguanosin (8-oxo-Gsn), an oxidative metabolite derived from RNA, in both plasma and urine samples of rhesus monkeys [202]. These findings revealed a positive correlation between aging and increased levels of these oxidation products in both blood and urine, with the most notable elevation observed in 8-oxoGsn in urine. Consequently, it is postulated that the presence of 8-oxoGsn in urine may serve as a potential biomarker for the process of aging, given the ease of obtaining urine samples [202]. In frail patients with CVD, the presence of urinary 8-oxoGsn was found to be independently associated with the development of mild cognitive impairment (MCI). The sensitivity and specificity of this association were 87.5% and 69.5%, respectively, suggesting that 8-oxoGsn may be a valuable indicator for the early detection of MCI risk in frail patients with CVD [203].

Detection of metabolites, whether in blood or urine, usually requires detection by mass spectrometry or NMR, which requires a higher level of technology and cost and is more difficult to promote clinically.

#### Recommendations

(1) Elevated levels of circulating CD8^+^CD28^-^ T-cell subsets have been associated with immune senescence and chronic inflammation, which in turn contribute to accelerated VA and increased risk of CVD (Level A evidence, Class IIa recommendation);
(2) Reduced levels of circulating EPCs are strongly associated with aging, endothelial injury, and endothelial dysfunction, making them reliable biomarkers of cumulative cardiovascular risk. However, the clinical promotion of EPC assessment is limited by their low abundance in the blood (Level A evidence, Class IIb recommendation);
(3) Numerous DNAmAge models have demonstrated the ability to accurately measure blood age across the lifespan, thereby influencing and reflecting VA. However, the clinical promotion of DNAmAge assessment is hampered by its technical complexity and high cost (Level A evidence, Class IIb recommendation);
(4) IGF-1, FGF21, Fibulin-1 and HERVK-Env proteins, ncRNAs, metabolites, EMPs, IgG glycosylation and DNA methylation in blood and metabolites in urine may be used as potential VA biomarkers but need to be further validated in subsequent cohort studies (Level C evidence, Class IIb recommendation).

### Building models to assess and predict VA

VA includes a range of changes in the vasculature at multiple levels, from the molecular to the functional. Consequently, the accurate and comprehensive assessment and prediction of VA and its associated diseases requires the integration of data derived from diverse markers operating across multiple scales, dimensions, and modalities, which also allows for a more consistent and universal VA assessment.

#### Models for assessing and predicting VA and VA-related diseases

One of the best-known aging assessment studies in healthy populations is the FHS [204]. The FHS is a rich, longitudinal, transgenerational, and deeply phenotyped cohort study that includes a number of studies on VA and VA-related diseases, such as the assessment of the degree of vascular endothelial damage and metabolic risk in individuals by measuring circulating EMP levels in the blood of community populations in conjunction with the FRS [162]; the assessment of aortic root remodeling by echocardiography, which tracks the diameter of the aortic root from adolescence to adulthood to determine the risk of aortic root remodeling in relation to CVD risk and outcomes [205]; and the cross-sectional study assessing the relationship between vascular stiffness indices, such as the cfPWV, and the accumulation of tau protein in regions of amyloid-β plaques in the brain, showing that targeting aortic stiffness could potentially serve as an independent approach for preventing tau-associated lesions in middle-aged and older adults without dementia [206].

To date, a multitude of healthy aging cohorts have been established around the world, including the China Health and Retirement Longitudinal Study [207], the Chinese Longitudinal Healthy Longevity Tracking Study [189], the Chinese Community-Dwelling People Study [208], and the US Health and Retirement Study [209], as well as in European countries [210], the United Kingdom [211], Republic of Korea [212], Mexico [213], India [214] and Costa Rica [215], among other studies focusing on health and aging cohorts. These collective endeavors contribute significantly to the global study of aging and promote VA studies.

Compared to aging research, VA research is still in its infancy. Currently, there is a lack of a well-developed and widely accepted assessment model for VA. However, the inclusion of VA indicators or risk factors in many existing aging assessment models has given these models the ability to predict VA and VA-related diseases to some extent. A recent study by Chen et al. in China exemplifies this by incorporating nine markers of aging representing different organ/system functions, including the vascular markers left humerus mean arterial pressure and baPWV, and using multiple linear regression, principal component analysis, and the Klemera-Doubal method (KDM) to develop a total of eight models for biological aging (BA) assessment, and among these models, one particular KDM model demonstrated potential suitability for evaluating BA in the Chinese Han population [216]. The use of the Levine clock, which incorporates clinical blood indicators such as blood glucose and CRP, enables it to reflect the impact of some CVD risk factors on BA and to better predict disease and death [172]. The GrimAge clock, which includes smoking and seven plasma proteins associated with inflammation, CVD, nephropathy, and cognitive function in addition to DNAmAge, has shown improved prognostic ability for disease onset, progression, and outcome [217]. By integrating cIMT and the PWV into an established FRS cardiovascular risk factor assessment model, a more accurate vascular aging index (VAI) can be derived, leading to improved predictive ability [218]. In addition, the National Health and Nutrition Examination Survey (NHANES) study incorporates diverse dietary and nutrition-related indicators, as well as vascular markers such as the ABI, enabling the assessment and prediction of CVDs such as PAD and atherosclerosis in patients with nonalcoholic fatty liver disease [219, 220].

In these health and aging models, the most commonly integrated vascular indices are systolic BP, diastolic BP, mean arterial pressure, PP, heart rate, PWV, cIMT, etc [221]. By incorporating these vascular indices along with CVD risk factors, these health and aging models provide a valuable framework for studying VA, provide a basis for exploring the mechanisms underlying VA, facilitate early detection of pathological VA, and serve as a basis for early intervention and the evaluation of effectiveness.

The advancement and utilization of big data, machine learning, and artificial intelligence have contributed significantly to the advancement of aging assessment. In addition, the integration of functional metrics, imaging data, and high-throughput data into aging assessment models is gradually being implemented. Since 2006, the FHS has progressively integrated various data sources, including genome-wide association study (GWAS) data, whole blood gene expression data, exon, microRNA, DNA methylation, and proteomics data [204]. However, the progressive incorporation of complex assessment metrics may make VA assessment more expensive and difficult to generalize. Therefore, an effective VA assessment model should include a comprehensive consideration of biomarkers and risk factors associated with VA, ranging from molecular to functional dimensions. In addition, redundant indicators with comparable or overlapping intrinsic mechanisms should be minimized to ensure that the overall scoring system is as accurate, concise, and balanced as possible. The model should also be user-friendly and easily adaptable for widespread implementation in the clinic.

From this point of view, it is imperative to allocate greater consideration to the prognostic significance of common scales, questionnaires, and simple clinical indicators of VA on a population scale. The American Heart Association (AHA) has recently revised and expanded its recommendations for cardiovascular health by introducing the “Life’s Essential 8 Score”, which consists of diet, physical activity, nicotine exposure, sleep health, body mass index, blood lipids, blood glucose, and blood pressure [222]. Each of these indicators, obtainable through questionnaires and simple clinical assessments, could independently predict cardiovascular risk and outcomes and may also predict the risk and outcomes associated with VA. Hence, the formulation of a concise and effective set of VA biomarkers, the establishment of a high-quality prospective cohort comprising VA patients and ensuring long-term follow-up, conducting epidemiological investigations to validate the clinical prognostic significance of these VA biomarkers, and advocating for the incorporation of these VA biomarkers into the guidelines for the management of VA are all urgent issues that need to be addressed.

#### Standardization of VA assessment

Numerous cohort studies on VA have been conducted worldwide, including in China, and various medical institutions have established their own diagnostic and treatment frameworks for this condition. However, there are discrepancies in the indicators included in different population cohort studies and medical institutions, as well as in the methods, medical instruments, and algorithms used for data collection and analysis. As a result, these discrepancies contribute to substantial diversity and inconsistency in the data produced across studies, thereby limiting the generalizability and scientific value of the data generated by individual institutions. Consequently, there is an urgent need to establish a cohesive and standardized framework for the diagnosis, treatment, data management, and research of VA. In pursuit of this goal, China has initiated the establishment of a Standardized Vascular Aging Management Center (VMC) at Tongji Hospital of Tongji Medical College of Huazhong University of Science and Technology (Wuhan, China), which will extend its reach to municipalities, counties, and communities nationwide, with the aim of facilitating the standardized management of vascular health in China and the development of assessment and intervention technologies for VA and VA-related diseases, as well as innovation and translation of VA research.

## Conclusion and future perspectives

**Figure 1.**
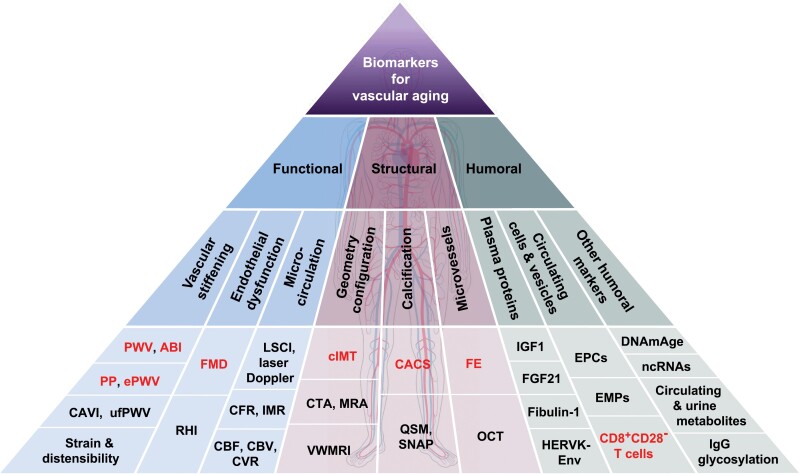
**Framework of biomarkers for vascular aging.** The proposed framework for vascular aging (VA) consists of three dimensions: functional, structural, and humoral biomarkers. The most recommended VA biomarkers (Table 3) shown in red letters cover multidimensional and multiscale changes in the vasculature during aging and could be widely used in routine clinical practice. The value of these VA biomarkers in assessing the biological aging of the vasculature needs to be further validated. The human circulation image is licensed for use by veer website. Abbreviations: ABI, ankle brachial index; CACS, coronary artery calcium scores; CAVI, cardio-ankle vascular index; CBF, cerebral blood flow; CBV, cerebral blood volume; CFR, coronary flow reserve; cIMT, carotid intima-media thickness; CTA, computed tomography angiography; CVR, cerebrovascular reserve; DNAmAge, DNA methylation age; EMPs, endothelial microparticles; EPCs, endothelial progenitor cells; ePWV, estimated pulse wave velocity; FE, fundoscopic examination; FGF21, fibroblast growth factor 21; FMD, flow-mediated dilation; HERVK-Env, human endogenous retroviruse K envelope protein; IGF-1, insulin-like growth factor 1; IgG, immunoglobulin G; IMR, index of microcirculatory resistance; LSCI, laser speckle contrast imaging; MRA, magnetic resonance angiography; PP, pulse pressure; OCT, optical coherence tomography; PWV, pulse wave velocity; QSM, quantitative susceptibility mapping; RHI, reactive hyperemia index; SNAP, simultaneous noncontrast angiography and intraplaque hemorrhage; ufPWV, ultrafast ultrasound imaging pulse wave velocity; VWMRI, vessel wall magnetic resonance imaging.

According to the advice from ABC experts, VA biomarkers were reviewed from three aspects of VA: functional, structural, and humoral (Fig. 1) [8]. Given their potential for broad clinical applicability, the most strongly recommended VA biomarkers include changes indicative of vascular stiffness (baPWV and ABI), endothelial function (FMD), vascular structure (cIMT and CACS), microvascular structure and distribution (fundus imaging), and proinflammatory factors (circulating CD8^+^CD28^−^ T cells; Table 3). The inclusion of the ePWV and PP, which reflect arterial stiffness in large and medium-sized arteries, could be justified by their reliance on blood pressure calculations without the need for additional measurements and their potential to aid in the assessment of VA (Table 3).

**Table 3.** Recommended biomarkers of vascular aging

Dimension	Biomarker	Test method	COR	LOE
Functional	baPWV	Vascular screening device	I	A
	FMD	Ultrasound	I	A
	ABI	Doppler measurement	I	A
	ePWV	Blood pressure measurement	IIa	B
	Pulse pressure	Blood pressure measurement	IIa	B
Structural	cIMT	Ultrasound	I	A
	Fundoscopic examination	Fundoscope	I	A
	CACS	Computed tomography	IIa	A
Humoral	Circulating CD8^+^CD28^−^ T cells	Flow cytometry	IIa	A

Abbreviations: ABI, ankle brachial index; baPWV, brachial-ankle pulse wave velocity; CACS, coronary artery calcium score; cIMT, carotid intima-media thickness; ePWV, estimated pulse wave velocity; FMD, flow-mediated dilation.

In subsequent studies, it is imperative to validate these markers in different age groups and to leverage high-quality population-based research on VA biomarkers, guided by this expert consensus. This will enable Chinese research teams to conduct more comprehensive investigations within the consortium’s framework, with effective implementation of consensus, collaboration, and knowledge sharing (Table 3, Fig. 1).

The action framework for VA biomarker research in China includes the following objectives: (i) to facilitate the establishment of a standardized and expanded cohort for the “1,000 Individuals Vascular Aging Research Program” within China; (ii) to identify and validate VA biomarkers that are appropriate for the Chinese population, with a focus on determining the critical point of VA and establishing the optimal intervention timeframe; (iii) to develop an artificial intelligence-based VA assessment system and a predictive model for VA-related diseases, along with the advancement of biomarker detection technologies; and (iv) to foster collaboration among industry, academia, research, and government to promote the establishment and implementation of research guidelines for VA biomarkers in China, with the ultimate goal of improving the vascular health of China’s elderly.
